# Actinorhizal plants and *Frankiaceae* : The overlooked future of phytoremediation

**DOI:** 10.1111/1758-2229.70033

**Published:** 2024-11-04

**Authors:** Ryan Michael Thompson, David George, Maria del Carmen Montero‐Calasanz

**Affiliations:** ^1^ School of Natural and Environmental Sciences, Newcastle University Newcastle upon Tyne UK; ^2^ IFAPA Las Torres‐Andalusian Institute of Agricultural and Fisheries Research and Training, Junta de Andalucía Seville Spain

## Abstract

Bioremediation of degraded soils is increasingly necessary due to rising food demand, reductions in agricultural productivity, and limitations in total available arable area. Several bioremediation strategies could be utilized to combat soil degradation, with phytoremediation emerging as a standout option due to its in situ approach and low implementation and maintenance costs compared to other methods. Phytoremediation is also a sustainable solution, which is increasingly desirable to blunt the progression of global warming. Actinorhizal plants display several desirable traits for application in phytoremediation, including the ability to revegetate saline soil and sequester heavy metals with low foliar translocation. Additionally, when grown in association with *Frankiaceae* endophytes, these abilities are improved and expanded to include the degradation of anthropogenic pollutants and the restoration of soil fertility. However, despite this significant potential to remediate marginalized land, the actinorhizal‐*Frankiaceae* symbiosis remains heavily understudied and underutilized. This review aims to collate the scattered studies that demonstrate these bioremediation abilities and explain the mechanics behind such abilities to provide the necessary insight. Finally, this review will conclude with proposed future directions for utilizing this symbiosis and how it can be optimized further to facilitate improved bioremediation outcomes.

## Graphical abstract

Actinorhizal plants, in symbiosis with Frankiaceae bacterial endosymbionts, possess traits beneficial for bioremediation. These plants can thrive in harsh conditions, such as high salinity and metal pollution, with their resilience enhanced by Frankiaceae. Additionally, Frankiaceae improve soil nutrients through nitrogen fixation and can degrade various anthropogenic pollutants.

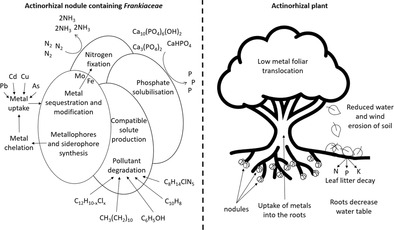

## INTRODUCTION

Bioremediation strategies are a class of “green technologies” that serve to restore degraded land, rendering it habitable and fit for arable use again. Being biologically based, such strategies are generally less intensive and polluting to the environment than physiochemical methods such as electrodialysis and solvent extraction among other examples (Sayqal & Ahmed, 2021). Bioremediation strategies can be divided into two broad classes, consisting of ex situ and in situ methods. Generally, in situ methods are preferable, as they are generally cheaper to implement, requiring less labour and space, as the bioremediation is conducted directly upon the polluted site, whereas ex situ methods consist of transporting the polluted material off site and treating it to remediate it (Azubuike et al., 2016).

In situ methods that introduce exogenous organisms for the purposes of bioremediation (enhanced in situ bioremediation) typically utilize microorganisms, with the exception of phytoremediation which instead utilizes plants (Azubuike et al., 2016; Sayqal & Ahmed, 2021). However, these plants can serve to aid the bioremediation effort alongside endemic soil microorganisms, with the efficacy of this approach also potentially bolstered through inoculation with desirable microorganisms.

Actinorhizal plants are noted pioneer species, well adapted to poor, disturbed soil, in part due to their symbiosis with the nitrogen fixing *Frankiaceae*. While *Frankiaceae* are noted as being capable of free‐living nitrogen fixation, through formation of oxygen excluding vesicles (Berry et al., 1993; Mohapatra et al., 2004). During symbiosis, less *Frankiaceae* resources may need to be devoted towards vesical formation, as the host plant is noted as contributing towards this micro‐oxic environment, in addition to the host providing *Frankiaceae* with photosynthesis derived carbon (Huss‐Danell, 1997; Persson & Huss‐Danell, 2008). In addition to this, as detailed in this review, this symbiosis, exhibits low foliar recycling of metals, effective revegetation of saline soil, degradation of anthropogenic pollutants and improvements to soil nutrients, making this symbiosis attractive for bioremediation. However, despite these desirable traits, the symbiosis remains heavily underutilized and understudied, with literature on this topic being scattered and difficult to locate.

Thus, this review aims to provide a cohesive overview of the actinorhizal‐*Frankiaceae* symbiosis to display its bioremediative potential. Such studies were located with relative ease through interrogation of databases such as PubMed searching key words related to the symbiosis and bioremediation, as the pool of literature from which to draw upon is relatively small. As such, the majority of literature detailing the actinorhizal‐*Frankiaceae* bioremediative capabilities has been considered. Critical review of these sources was warranted to accurately display the bioremediative capabilities of this symbiosis, especially as the limited numbers of studies considered in this review utilized differing methodologies and considered differing plant growth metrics and soil chemistry metrics. In addition, the conclusions reached by some studies differed from each other, therefore critical analysis is warranted to account for such differences. However, prior to discussion of the actinorhizal‐*Frankiaceae* symbiosis, the sources and effects of soil degradation will be briefly introduced.

### Sources and effects of soil degradation

Soil degradation is a natural process to a degree, with volcanic activity, forest fires and rock weathering releasing heavy metals into the soil, with soil salinization driven naturally by evaporation of water from soil and deposition of salts through rock weathering (Hassanien & Shahawy, 2010; Rengasamy, 2006). Nevertheless, anthropogenic activity has greatly increased the rate and scope of these processes (Lal, 2015).

### Soil pollution

Heavy metals and other polluting chemicals are released into the environment during energy production, mining, manufacturing and waste disposal, among other activities (Adesokan et al., 2016; Hassanien & Shahawy, 2010; Nicholson & Chambers, 2008; Yang et al., 2018; Zhang, Wu, & Simonnot, 2012), leading to soil contamination. Agricultural activities further soil pollution due to wastewater irrigation and use of pollutant‐ and metal‐containing agrochemicals (Barker & Gimingham, 1911; Mahfooz et al., 2020; Nicholson & Chambers, 2008; Sager, 2007; Sayo et al., 2020). These anthropogenic activities have increased the number of polluted sites across Europe (and presumably the rest of the world), with the majority of these sites remaining unremediated (EEA, 2022; Van Liedekerke et al., 2014).

In terms of effects upon agriculture, heavy metals cause oxidative damage which hinders crop growth (Opdenakker et al., 2012; Pourrut et al., 2008). This growth inhibition results from heavy metal stress leading to reduced photosynthetic pigment concentration, inhibition of enzymes involved in photosynthetic and other key plant production processes (Dhir et al., 2011; Di Salvatore et al., 2008; Li et al., 2005; Rivetta et al., 1997; Sridhar et al., 2014).

In addition to impacts upon agricultural productivity, heavy metal pollution can lead to serious medical conditions such as Minamata and Itai‐itai disease and a range of other health effects, with chronic exposure linked to cancer development (Aoshima, 2016; Gebeyehu & Bayissa, 2019; Harada, 1995; Khan et al., 2008; Sayo et al., 2020; Zhou et al., 2016). Whilst some studies indicate that crops grown upon metal polluted soils pose little risk to consumers (Khan et al., 2008), results presented by Zhuang et al. (2009), Balkhair & Ashraf, 2016, and Gebeyehu and Bayissa (2019) present these metals exceeding safety thresholds in crops grown upon metal polluted sites (including cancer risk thresholds).

Aside from crops, seafood and drinking water may also be polluted by heavy metal run off from contaminated soil (Gupta et al., 2009; Islam et al., 2015; Kobayashi et al., 2009; Nriagu et al., 1979; Taboada‐Castro et al., 2012; von Gunten et al., 1997). Although many studies indicate these food sources are safe for moderate consumption (Djedjibegovic et al., 2020; Maurya et al., 2019; Sobhanardakani, 2017; Sobhanardakani et al., 2018). These studies often only consider metals individually, when considered together using metrics such as the “total health risk index” (and its analogues), the resulting cumulative exposure values indicate risk to human health (El‐Shenawy et al., 2016).

In addition to heavy metals, soil can become polluted from a range of anthropogenic generated compounds. Those pertinent to the actinorhizal‐*Frankiaceae* symbiosis including atrazine, biphenyl compounds, hydrocarbons and phenolic compounds, will be detailed in this review. In the case of atrazine, this pollutant is released into the environment as it is applied to crops as a broadleaf herbicide. Despite atrazine being banned in Europe in 2004, it is still extensively used, with an estimated 70 million tonnes applied to crops annually in America (US geological survey, 2012). This heavy usage is problematic as the half‐life of atrazine can be up to 742 days, depending upon environmental conditions, potentially allowing accumulation in the environment and food chains (Solomon et al., 1996; Zhang et al., 2014). It is of interest to reduce environmental atrazine levels due to its potential teratogenic, endocrine and immune system disrupting effects (Jablonowski et al., 2011; Rohr & McCoy, 2010; Solomon et al., 1996).

Biphenyl compounds such as polychlorinated biphenyl (PCB), have been used as coolant and insulating fluids in electrical equipment, hydraulic lubricants and applied as plasticisers (Nisbet & Sarofim, 1972). PCBs exhibit a range of negative health effects including carcinogenicity, endocrine disruption, immunosuppression, reproductive and developmental issues, with many of these seen in the Japanese Yusho poisoning (Aoki, 2001; Geusau et al., 2001; Hagmar et al., 2001; Jacobson & Jacobson, 1996; Kramer et al., 2012; Kuratsune et al., 1972; Lauby‐Secretan et al., 2013; Masuda, 2003; Mocarelli et al., 2000; Schell et al., 2014; Stewart et al., 2000; Tsukimori et al., 2008; Yoshimura, 2012). Due to these negative health effects PCBs were banned in 2001, despite this it is estimated that 80% of PCBs are still present within the environment (Othman et al., 2022). Such pollution arises due to volatilization of PCBs disposed of within landfill, leaks from electrical transformers, incineration of PCB containing waste, improper disposal of PCB waste and accidental release (Duke et al., 1970; Kuratsune et al., 1972; Othman et al., 2022; Yoshimura, 2012).

Hydrocarbons are released in the environment most obviously through spills, such as the Deepwater Horizon oil spill which released an estimated five million barrels, and oil fires such as those seen in the 1991 Gulf War (McNutt et al., 2012; Yihdego & Al‐Weshah, 2017). Other sources of hydrocarbon pollution are incomplete combustion of organic materials such as coal and oil, and industrial manufacturing (Abdel‐Shafy & Mansour, 2016). Hydrocarbons are associated with damage to the central nervous, gastrointestinal, renal, hepatic, immune and cardiovascular systems, and are potentially carcinogenic and teratogenic (Abdel‐Shafy & Mansour, 2016; ATSDR, 1999; Marris et al., 2020; Perera et al., 2012; Rengarajan et al., 2015; Shiue, 2016; Tong et al., 2018; Zheng et al., 2018).

Phenol pollution arises from a range of sources including pulp and paper mills, petroleum and coal refining, petrochemical manufacture, pharmaceuticals and tanneries (Kumaran & Paruchuri, 1997; Lindström & Nordin, 1976). Phenol compounds are associated with a range of negative health effects in humans, such as nausea, vomiting, diarrhoea, abdominal pain, and a burning sensation in the oral cavity/pharynx, with more severe phenol poisoning being fatal (Boatto et al., 2004; Jarvis et al., 1985; Kim et al., 1994; Philip & Marraffa, 2012). Animal studies suggest phenol exposure may also lead to chronic issues with the nervous, cardiovascular, hepatic, renal, and immune systems; however, human studies regarding phenol are limited, usually with compounding factors (ATSDR, 2008; EPA, 2000; PHE, 2016). Although phenol cannot definitively be said to cause long term adverse medical effects in humans, removal of these pollutants from the environmental is still recommended based upon their acute toxicity and potential to cause chronic effects.

### Salinity

Increasing soil salinity presents challenges to land use, with 10 million hectares of land being abandoned annually due to high salinity and 50% of arable land predicted to be salt affected by 2050 (Szabolcs, 1989; Wang et al., 2003). Anthropogenic activity increases soil salinity due to irrigation, which raises water tables bringing salts closer to the soil surface, the use of saline irrigation water and the use of salts as de‐icing agents on roads (Chen et al., 2010; Dobson, 1991; Ma et al., 2008; Oosterbaan, 1988; Pang et al., 2010; Rengasamy, 2006).

Salinity contributes to reductions in agricultural productivity as high salt levels decrease soil water potential, making water and nutrients more difficult for plants to acquire (Cruz et al., 2018; Hu & Schmidhalter, 2005). Furthermore, salinity reduces stomatal and mesophyll conductance, reducing CO_2_ diffusion, overall impairing photosynthesis (Delfine et al., 1998; Delfine et al., 1999). Reductions in photosynthesis subsequently lead to generation of reactive oxygen species, causing deleterious oxidative damage, further inhibiting plant growth (Ali et al., 2004; Dobson, 1991; Ozgur et al., 2013; Singh et al., 2012). Reductions in growth are exemplified by many studies which show salt stressed crops performing poorer in a range of metrics compared to unstressed crops (Abbas et al., 2013; Magán et al., 2008).

### Soil nutrient depletion

In an agricultural environment soil becomes nutrient depleted over time as crops are removed at harvest, necessitating replacement of the lost macronutrients and micronutrients with chemical fertilizers to maintain productivity. This cannot be avoided, however, crop management practices which leave minimal post‐harvest residual material (such as stover and straw), may lead to greater losses of nutrients than necessary (Blanco‐Canqui & Lal, 2009; Karlen et al., 1994; Salinas‐Garcia et al., 2001).

To alleviate the effects of reduced soil nutrients, economically and energetically expensive fertilizers are applied to soil. Such fertilizers are estimated to contribute 1–2% of global CO_2_ emissions, acting as a driver of climate change, in turn hampering agricultural output (Mbow et al., 2019; Walling & Vaneeckhaute, 2020; Xx, 2023). In addition, fertilizers can leach into nearby water bodies causing eutrophication, with the resulting algal blooms depleting the water of oxygen and potentially producing toxins (Anderson, 1994; Carpenter et al., 1998; Flewelling et al., 2005; Gilbert et al., 2006; Jeglitsch et al., 1998; Kotak et al., 1993; Poli et al., 1986; Tong & Chen, 2002; Watkins et al., 2008).

## BIOREMEDIATION STRATEGIES

### In situ bioremediation

As detailed earlier, in situ methods are preferable, with the most basic of these being known as natural attenuation which involves leaving the site for indigenous organisms to restore through natural biological processes. In some cases this process is enhanced through the use of bioinoculants (bioaugmentation), whereas in other cases may stimulate aerobic degradation by the native soil microflora by introducing oxygen into the soil (bioventing) or by increasing oxygen concentration in the groundwater (biosparging). Such enhanced in situ bioremediation methods are expected to show greater bioremediation rates, particularly in the case of bioaugmentation as the introduced organisms have been specifically selected for their bioremediative properties (Azubuike et al., 2016; Sayqal & Ahmed, 2021).

### Phytoremediation and microbially‐assisted phytoremediation

Regarding in situ approaches, phytoremediation is generally viewed as one of the cheaper options in contrast to other methods, which may require acquisition, installation of expensive equipment. Phytoremediation instead utilizes plants to remove pollutants from the soil, presenting a low cost regarding acquisition and planting of the plants, with inputs maintenance as once established plants are generally self‐sufficient (Wang & Delavar, 2023). However, phytoremediation efficacy may be limited by physiological constrains, most notably slow growth which limits turnaround time, small root systems which limit access to deeper pollutants, difficulty in accumulating certain non‐bioavailable metals and poor ability to survive in heavily polluted soils (Adeoye et al., 2021; Sayqal & Ahmed, 2021; Wang & Delavar, 2023).

Some of these constraints such as slow growth are intrinsic to the nature of plants, so phytoremediation may be best applied to sites in which a long‐time horizon is envisaged for the bioremediation process (Wang & Delavar, 2023). Other limitations can be alleviated through careful selection of the plants used, with actinorhizal plants being a particularly attractive example as they exhibit rapid growth upon inhospitable soils while also accumulating metals. In a similar manner to bioaugmentation, microbial inoculants can be deployed alongside plants to further lessen some of the challenges associated with phytoremediation, with this approach known as microbially associated phytoremediation.

Microbes have been shown to aid phytoremediation by forming symbioses with plants, improving growth under stress conditions, enhancing pollutant uptake and degradation of contaminants the host plant is unable to. Successful examples include the supplementation of Alfalfa with *Pseudomonas aeruginosa*, which increased the rate of petroleum hydrocarbon removal compared to the plants or microbes alone, while also improving plant growth compared to uninoculated plants (Agnello et al., 2016). Similar results have also been observed for other contaminants including 2,4‐dichlorophenoxyacetic acid, phenanthrene, 2‐chlorobenzoic acid, among other examples (Germaine et al., 2006; Sheng & Gong, 2006; Siciliano & Germida, 2009). As such, plants with desirable characteristics and the capability of forming associations with symbionts which can assist in phytoremediation are highly desirable. Actinorhizal plants and their *Frankiaceae* endosymbionts could represent such a symbiosis, offering significant potential in microbially assisted phytoremediation.

## ACTINORHIZAL PLANTS AND THEIR ENDOSYMBIONT

### Actinorhizal plants

Actinorhizal plants are a group of rosid plants consisting of eight families of perennial angiosperms, all of which are trees or shrubs (except those in the *Datisca* genus) (Dawson, 2008; Doyle, 2011). Actinorhizal plants are pioneer species found globally in a range of often ecosystems, often occurring in inhospitable, nutrient poor environments such as deserts and costal dunes (Dawson, 1986; Dawson, 2008).

The ability of actinorhizal plants to grow in inhospitable sites is partly due to their root symbiosis with nitrogen‐fixing *Frankiaceae*, with 280 of the 420 actinorhizal species displaying this association (Dawson, 2008). This symbiosis is primarily driven by intra‐ or intercellular infection of the host plant roots, with this infection stimulating nodule primordium formation through modification of lateral root cells (Pawlowski, 2008). As detailed earlier, the nodule environment, contributes to creation of a micro‐oxic conditions while also supplying carbon to *Frankiaceae*, facilitating *Frankiaceae* growth and nitrogenase function (Huss‐Danell, 1997; Persson & Huss‐Danell, 2008).

Such a symbiosis has been noted as providing 61% of the nitrogen requirement for a stand of *Ceanothus velutinus* (Dawson, 2008; Zavitkovski & Newton, 1968). Demonstrating why inoculation of actinorhizal plants with *Frankiaceae* often significantly increases host plant growth. Examples of such improvements to host plant growth include inoculation of *Casuarina equisetifolia* with *Frankiaceae* strains CeFr1 and Cefr2 which yielded a positive change in a range of growth metrics after just 90 days, significantly increasing plant height, stem girth and survival after 2 years in field conditions (Karthikeyan, 2016). Likewise, inoculation of *Alnus* resulted in greater total dry weight and nitrogen content compared to uninoculated trees within 6 months of planting (Wheeler et al., 1991).

It is estimated the actinorhizal‐*Frankiaceae* symbiosis fixes 240–350 kg ha^−1^ of nitrogen per year (Wall, 2000). In some instances this may even be exceeded, as it has been shown a 15‐year‐old *Ceanothus velutinus* stand added 432 kg ha^−1^ of nitrogen to the soil by deposition of leaf litter alone (Zavitkovski & Newton, 1968). Conversely, this figure may also be drastically lower under unfavourable environmental conditions and poor forestry management, thought this is relatively uncommon (Dommergues, 1997). Such nitrogen rich leaf litter has been noted as improving growth of other plant species, exemplified by conifer forests containing *A. rubra* exhibited increased soil nitrogen compared to conifer monocultures, due to decomposition of *A. rubra* leaf litter (Tarrant et al., 1969). This is desirable for bioremediation as this will aid in restoration of soil fertility and reforestation.

### Frankiaceae

*Frankiaceae* is a poorly studied family of Gram‐positive, difficult to cultivate actinobacteria, which grows primarily through filamentous vegetative hyphae, producing vesicles and intercalary or multilocular sporangia. *Frankiaceae* are symbionts of actinorhizal plants but have also been found free‐living in soil, devoid of hosts (Benson & Silvester, 1993; Maunuksela et al., 1999; Ridgway et al., 2004).

The family *Frankiaceae*, within the order *Frankiales* (Sen et al., 2014) within the phylum Actinomycetota, was proposed in 1970 (Becking, 1970), initially solely containing the *Frankia* genus (Brunchorst, 1886). All *Frankiaceae* species were initially grouped into four clusters, with these clusters elevated to genus level in 2022 and named *Parafrankia*, *Protofrankia* and *Pseudofrankia* in addition to *Frankia* (Gtari, 2022) (Table 1).

**TABLE 1:** Validly named and candidatus species within the *Frankiaceae* family, their infectivity ranges and strains belonging to each species (Gtari et al., 2020; Herrera‐Belaroussi et al., 2020; Nguyen et al., 2019; Normand et al., 2023; Normand & Fernandez, 2019; Nouioui, Ghodhbane‐Gtari, Jando, et al., 2023; Nouioui, Ghodhbane‐Gtari, Pötter, et al., 2023; Nouioui, Neumann‐Schaal, Pujic, et al., 2023; Pozzi et al., 2020), with type species indicated in bold. The “*” symbol indicates this genus is infective to *Casuarinaceae* except *Gymnostoma*. While *Frankia nepalensis* is classified as a *Frankia* species, it is believed this was done under the old four cluster classification system rather than the new proposed genera (Gtari, 2022). This is based upon *F. nepalensis* exhibiting traits associated with *Pseudofrankia* and being most closely related to species within this genus, thus *F. nepalensis* will be considered a *Pseudofrankia* for the purposes of this review.

Genus	Species within the genus (strains of each species)	Infectivity range
*Frankia*	*Frankia alni* (**ACN14a** ^**T**^, AvcI1, MpI1, M16467, M16477)	*Alnus*
	*Frankia canadensis* (**ARgP5** ^**T**^)	Casuarinaceae*
	*Frankia casuarinae* (**CcI3** ^**T**^, ORS020606 (CeD), ORS022602, ORS020607 (CeF), ORS020608, HFP022801 (AllI1), ORS021001 (Cj1‐82), ORS02060, CcI2, BMG5.23, Thr, CeD, TA, Cj1‐82, Cg70.4, Allo2, CcI6, BR, Cg70.3, ORS022602, CeF, ORS020608, AllI1, ORS020609)	*Myricaceae*
	*Frankia gtarii* (**Agncl‐4** ^**T**^, Agncl‐10)	
	*Frankia tisai* (**Agncl‐8** ^**T**^, Agncl‐18)	
	*Frankia torreyi* (**CpI1** ^**T**^, CoN24d, ACN1Ag, Ag24‐251, ArI3, ARgN22d, Ar24H3, Ar24O2, A2J)	
	*Frankia umida* (**Ag45/Mut15** ^**T**^)	
	*Candidatus* Frankia alpina (**AiOr** ^**T**^, AvVan)	
	*Candidatus* Frankia nodulisporulans (**AgTrS** ^**T**^, AgUmASt1 and AgUmASH1)	
*Parafrankia*	*Parafrankia colletiae* (**Cc1.17** ^**T**^)	*Colletieae*
	*Parafrankia elaeagni* (**BMG5.12** ^**T**^)	*Elaeagnaceae*
	*Parafrankia discariae* (**BCU110501** ^**T**^)	*Gymnostoma*
	*Parafrankia irregularis* (**G2** ^**T**^)	*Myricaceae*
	*Parafrankia soli* (**Cj** ^**T**^)	
*Protofrankia*	*Protofrankia coriariae* (**BMG5.1** ^**T**^)	*Coriariaceae*
	*Candidatus* Protofrankia californiensis (**Dg2** ^**T**^)	*Datiscaceae*
	*Candidatus* Protofrankia datiscae (**Dg1** ^**T**^)	*Dryadoideae*
	*Candidatus* Protofrankia meridionalis (**Cppng1_Ca_nod** ^**T**^)	*Ceanothus*
*Pseudofrankia*	*Pseudofrankia asymbiotica* (**M16386**)	Unable to reinfect host plants
	*Pseudofrankia inefficax* (**EuI1c** ^**T**^)	
	*Pseudofrankia saprophytica* (**CN3** ^**T**^)	
	*Frankia nepalensis* (**CN4** ^**T**^, CN6, CN7, CNm7)	

Despite *Frankia* first being described by Woronin (1866), the type species *Frankia alni* was not validated until 2016, due to difficulty in locating a strain that matched the original description (Nouioui et al., 2016). The proposal of the type strain has since led to a large boom in the classification of *Frankiaceae* species (Gtari, 2022). Currently 17 validly named *Frankiaceae* species have been proposed, alongside five candidatus species (Table 1).

## *FRANKIACEAE*‐ACTINORHIZAL BIOREMEDIATIVE ABILITIES

### Frankiaceae*‐actinorhizal tolerance to salinity and salinity reduction*

**FIGURE 1:**
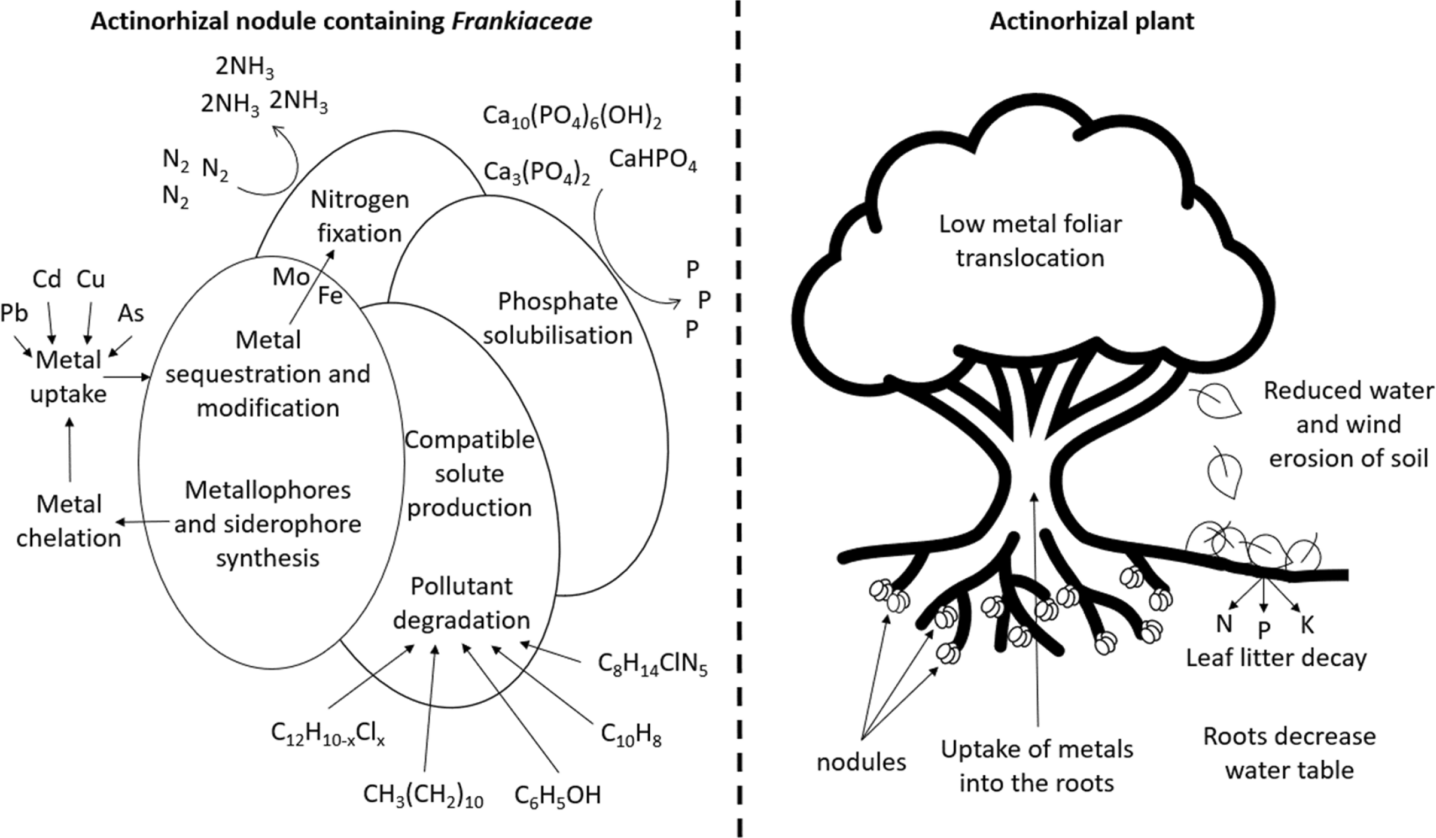
Diagrammatic representation of the bioremediative capabilities of the *Frankiaceae*‐actinorhizal symbiosis. Left side of the image shows the abilities of the *Frankiaceae* within the nodule and the right side of the image shows the bioremediative abilities of the actinorhizal plants.

Revegetation of saline sites can reduce further salinization and potentially reduce existing salinity. This occurs as revegetation reduces wind and water soil erosion and lowers the water table by intercepting rainfall and increasing water uptake by the plants (Schofield & Scott, 1991) (Figure 1). Actinorhizal plants may facilitate revegetation of saline sites, with the *Frankiaceae* symbiosis improving their survival and in turn, their ability to reduce salinity (Table 2).

**TABLE 2:** Studies related to bioremediation that utilize *Frankaiceae* strains and actinorhizal plants. These studies are grouped in the below regarding which aspect of bioremediation they are concerned with. In cases where only *Frankiaceae* species or actinorhizal plants species were utilized in the study this has been noted with the phrase not utilized listed under the corresponding column of the table. The names of the *Frankiaceae* species and strains used in the studies detailed in the below table have been updated to include the species name where possible.

Actinorhizal plant species used	*Frankiaceae* species used	Reference
**Salinity remediation**		
*Casuarina glauca*	*Frankia* sp. CcI156, *Frankia* sp. CgIM4	Mansour et al., 2016
*Casuarina glauca, Casuarina equisetifolia*	*Frankia* casuarinae CcI3, *Frankia* casuarinae CeD	Ngom et al., 2016
*Elaeagnus macrophylla*	*Frankia* sp. Ema1	Tani & Sasakawa, 2000
*Casuarina equisetifolia*	*Frankia* sp. Ceq1	Tani & Sasakawa, 2003
*Elaeagnus angustifolia*	Not utilized	Qi et al., 2018
*Elaeagnus angustifolia*	Not utilized	Khamzina et al., 2006
*Alnus glutinosa*	Not utilized	Deptuła et al. (2020)
Not utilized	*Frankia casuarinae* CcI6	Oshone et al., 2013
Not utilized	*Frankia casuarinae* Allo2, *Frankia casuarinae* CcI6, *Frankia casuarinae* Thr, *Frankia casuarinae* CeD, *Frankia casuarinae* CcI3, *Frankia casuarinae* BMG5.23, *Frankia casuarinae* CgI82, *Frankia casuarinae* BR *Frankia* sp. DC12, *Frankia alni* ACN14a *Frankia inefficax* EuI1C, *Frankia* sp. EAN1pec	Oshone et al., 2017
Not utilized	*Frankia alni* ACN14a	Ghedira et al. (2017)
*Casuarina glauca, Casuarina equisetifolia*	*Frankia casuarinae* CcI3, *Frankia casuarina* CeD	Ngom et al., 2016
*Casuarina glauca*	*Frankia* sp. Thr	Batista‐Santos et al., 2015
*Casuarina glauca*	*Frankia* sp. Thr	Duro et al., 2016
**Heavy metal bioremediation**		
*Alnus glutinosa*	Not utilized	Mertens et al., 2004
*Alnus glutinosa*, *Alnus incana*	Not utilized	Lorenc‐Plucińska et al., 2013
*Alnus glutinosa*	*Frankia alni* ACN14a	Bélanger et al., 2015
*Alnus glutinosa*	*Frankia* sp. UGL 010708, *Frankia* sp. UFI 010708, *Frankia* sp. UFI 13270238	Wheeler et al., 2001
*Alnus incana*	Not utilized	Rosselli et al., 2003
*Alnus nepalensis*	Not utilized	Jing et al., 2014
*Casuarina glauca*	*Frankia* sp. BMG5.22, *Frankia casuarinae* BMG5.23	Ghazouani et al., 2020
*Alnus crispa*, *Alnus glutinosa*	*Frankia* alni AvcI1	Callender et al., 2016
*Alnus glutinosa*	*Frankia* sp. WgAvcI1	Pawlowski et al., 1997
Not utilized	Not utilized	Gupta et al., 2002
*Alnus glutinosa*	Not utilized	Vandecasteele et al., 2008
*Alnus glutinosa*	Not utilized	Desai et al. (2019)
*Alnus hirsute*, *Alnus firma*	Not utilized	Lee et al., 2009
Not utilized	*Frankia* torreyi ACN1^AG^, *Frankia* casuarinae CcI3, *Parafrankia* colletiae Cc1.17, *Pseudofrankia* saprophytica CN3, *Frankia torreyi* CpI1‐S, *Frankia torreyi* CpI1‐P, *Frankia* sp. DC12, *Frankia* sp. EI5c, *Frankia* sp. EAN1pec, *Pseudofrankia inefficax* EuI1c, *Frankia* sp. EUN1f, *Frankia* sp. QA3	Richards et al., 2002
Not utilized	*Frankia inefficax* EuI1c, *Frankia saprophytica* CN3, *Frankia alni* ACN14a, *Frankia casuarinae* CcI3	Rehan et al., 2019
Not utilized	*Frankia casuarinae* CcI3, *Frankia alni* ACN14a, *Frankia* sp. QA3, *Frankia* sp. EUN1f, *Frankia* sp. EAN1pec, *Pseudofrankia inefficax* EuI1c, *Pseudofrankia saprophytica* CN3, *Frankia* sp. DC12	Furnholm & Tisa, 2014
Not utilized	*Pseudofrankia saprophytica* CN3, *Frankia* sp. DC12, *Pseudofrankia inefficax* EuI1c	Rehan, Furnholm, et al., 2014
Not utilized	*Frankia* sp. ACN10a, *Frankia* sp. ACN12a, *Frankia* alni ACN14a, *Frankia* casuarinae CcI3, *Frankia* sp. CH37, *Frankia* torreyi CPI1, *Frankia casuarinae* Cg70.4, *Frankia* sp. Cg70.9, *Frankia casuarinae* Cj1‐82, *Frankia* sp. Ea1‐12, *Frankia* sp. DC12, *Parafrankia discariae* BCU110501, *Frankia* sp. DSMZ 44251	Deicke et al., 2019
Not utilized	*Frankia* sp. CH37	Mohr et al., 2021
*Casuarina equisetifolia*	Not specified	Karthikeyan et al., 2009
*Alnus glutinosa*	Not utilized	Kuznetsova et al., 2011
**Anthropogenic pollutant remediation**		
Not utilized	*Frankia alni* ACN14a, *Pseudofrankia inefficax* EuI1c	Rehan, Kluge, et al., 2014
Not utilized	*Pseudofrankia inefficax* EuI1c, *Frankia* sp. EUN1f, *Frankia* sp. CcI49, *Parafrankia irregularis* G2, *Frankia* sp. R43	Mansour et al., 2017
*Alnus glutinosa*	Not utilized	Kuznetsova et al., 2010
*Casuarina equisetifolia*	Not utilized	Sun et al., 2004
*Alnus crispa*	*Frankia alni* AvcI1	Lefrançois et al., 2010
*Alnus viridis* ssp. crispa, *Alnus incana* ssp. rugosa	*Frankia alni* AvcI1	Bissonnette et al., 2014
Not utilized	*Frankia* sp. QA3, *Frankia* alni ACN14a, *Frankia* sp. EAN1pec, *Pseudofrankia inefficax* EuI1c, *Frankia* sp. EUN1f, *Parafrankia elaeagni* BMG5.12	Rehan et al., 2016
**Soil nutrient improvement and plant growth promotion**		
*Ceanothus Velutinus*	Not specified	Zavitkovski & Newton, 1968
*Casuarina equisetifolia*	*Frankia* sp. CeFr1, *Frankia* sp. CeFr2	Karthikeyan, 2016
*Alnus rubra, Alnus glutinosa*	*Frankia* sp. Ar 1.2.5q, *Frankia* sp. ArI4, *Frankia* sp. Ag 1.1.8	Wheeler et al., 1991
*Alnus rubra*	Not specified	Tarrant et al., 1969
Not utilized	*Frankia alni* ACN14a, *Frankia torreyi* CPI1, *Frankia torreyi* ACN1^AG^, *Frankia* sp. QA3, *Frankia casuarinae* CcI3, *Frankia casuarinae* Allo2, *Frankia casuarinae* BMG5.23, *Frankia casuarinae* CcI6, *Frankia casuarinae* CeD, *Frankia casuarinae* Thr, *Frankia coriariae* BMG5.1, *candidatus* Frankia datiscae Dg1, *Frankia elaeagni* BMG5.12, *Frankia discariae* BCU110501, *Frankia* sp. EUN1f, *Frankia* sp. EAN1pec, *Frankia irregularis* DSM 45899, *Frankia* sp. R43, *Frankia saprophytica* CN3, *Frankia inefficax* EuI1c, *Frankia* sp. DC12	Nouioui, Cortés‐albayay, et al., 2019
Not utilized	*Frankia torreyi* CpI1	Nouioui, Ghodhbane‐Gtari, et al., 2019
Not utilized	*Frankia canadensis* ARgP5	Normand et al., 2018
Not utilized	*Frankia* sp. RT, *Frankia* sp. Rif, *Frankia casuarinae* Thr, *Frankia* sp. BR, *Parafrankia irregularis* G2, *Parafrankia soli* Cj, *Frankia* sp. URU, *Frankia* sp. CH, *Frankia* sp. G82	Arahou et al., 1998
*Casuarina equisetifolia*	Not utilized	Mailly & Margolis, 1992
*Casuarina equisetifolia*	Not specified	Parrotta, 1999
*Casuarina equisetifolia*	Not utilized	Izquierdo et al., 2005
*Elaeagnus angustifolia*	Not utilized	Khamzina et al., 2006
*Elaeagnus angustifolia*	Not utilized	Qi et al., 2018
*Alnus incana*	Not utilized	Taylor et al., 1989
Not utilized	*Frankia* sp. DDNSF‐01, *Frankia casuarinae* DDNSF‐02	Marappa et al., 2020
*Alnus incana*	Not utilized	Uri et al., 2001
Not utilized	*Frankia* sp. 52065, *Frankia* sp. HFPCcI3, *Frankia* sp. HFPArI3, *Frankia* sp. ArI5, *Frankia* sp. HFPCPI1, *Frankia* sp. AvsI3, *Frankia* sp. Air11, *Frankia* alni AvcI1	Aronson & Boyer, 1994
Not utilized	*Frankia* sp. AiPs1	Haansuu et al., 2001
Not utilized	*Frankia* sp. AiPs1	Klika et al., 2001
Not utilized	*Frankia* sp. AiPs1	Klika et al., 2003
Not utilized	*Frankia* alni ACN14, *Frankia casuarinae* CcI3, *Frankia* sp. EAN1pec	Udwary et al., 2011
Not utilized	*Frankia* sp. HFPArI3, *Frankia* sp. HFPCPI1, *Frankia* sp. HFPCcI2, *Frankia* sp. HFPCcI3, *Frankia* sp. HFPGpI1	Safo‐Sampah & Torrey, 1988
*Casuarina equisetifolia*	*Frankia* sp. UMCe12, *Frankia* sp. UMCe23, *Frankia* sp. UMCe35, *Frankia* sp. UMCe55	Gopinathan, 1995
Not utilized	*Frankia* alni AvCI1	Wheeler et al., 1984
*Ochetophila trinervis*	*Parafrankia discariae* BCU110501	Solans et al., 2011
*Alnus rubra*	*Frankiaceae* sp. HFPArI3	Berry et al., 1989
Not utilized	*Frankiaceae* sp. HFPArI3	Stevens & Berry, 1988

The salinity tolerance of actinorhizal plants still generates some controversy, with studies such as Mansour et al. (2016) suggesting that species such as *Casuarina glauca* are not saline tolerant, as these plants are unable to survive in NaCl concentrations greater than 200 mM. Other studies such as Ngom et al. (2016) instead state that *C. glauca* is saline tolerant. Such discrepancies between studies may be explained by osmotic shock, as the NaCl was added to the planting media more rapidly in Mansour et al. (2016) compared to others. Therefore, such methodological nuances should be considered when reviewing plants for use in saline environments.

Aside from *Casuarinaceae*, *Elaeagnaceae* is noted as being suitable for bioremediation of saline sites but displays a lower saline tolerance than *Casuarinaceae*. Seed germination of *Elaeagnus macrophylla* decreased sharply in concentrations of NaCl higher than 50 mM, with no germination occurring at 200 mM (Tani & Sasakawa, 2000). However, *E. macrophylla* seedlings showed minimal decrease in biomass at increasing NaCl concentration, indicating that growth may be less saline affected than germination. Examples of *E. macrophylla* use for treatment of saline sites include plantation upon areas of the Yellow River Delta (Qi et al., 2018). Such plantation resulted in reductions in soil salinity while increasing nitrogen, phosphorus and potassium compared to unplanted soil, with salinity reduction and nutrient accumulation increasing each year. In another study, *Elaeagnus angustifolia* was planted upon saline soil (3.3–4.3 dSm^−1^) around the Aral sea, with 96–100% of these plants surviving 19 months after planting and exhibiting rapid root and aerial tissue growth (Khamzina et al., 2006). Although not measured, such plant growth would be expected to reduce salinity by limiting water loss from the soil, lower the water table and improve the soil structure.

*Alnus glutinosa* may also be useful in revegetation of saline sites, particularly in boreal environments in which *Casuarina* and *Elaeagnus* are not suited. While *A. glutinosa* is well known for its ability to thrive in waterlogged sites, there has been some conflict regarding its salinity tolerance (Claessens et al., 2010). For example, studies such as Gómez Mercado et al. (2012) and Dirr (1976) suggest that *A. glutinosa* does not have good saline tolerance, while Dobson (1991), Mertens et al. (2004), and Deptuła et al. (2020) report moderately good saline tolerance in *A. glutinosa*. Similarly to *Elaeagnaceae*, *A. glutinosa* may be more susceptible to salinity during germination and early growth stages, so transplantation of plants after germination to saline sites may facilitate their revegetation with *Alnus* (Deptuła et al., 2020).

It is well known that certain endophytic bacteria can improve plant tolerance to abiotic stress, with *Frankiaceae* capable of eliciting such a response in actinorhizal plants (Kamran et al., 2022). *Frankiaceae* strains have displayed instances of high salinity tolerance, with strains such as CcI6 and Allo2 displaying a minimum inhibitory concentration of 1000 mM to NaCl (Oshone et al., 2013; Oshone et al., 2017). However, others such as Ceq1 and CcI3 present lower minimum inhibitory concentrations of 500 and 475 mM NaCl, respectively (Oshone et al., 2017; Tani & Sasakawa, 2003). When comparing the genomes of salt tolerant strains to less tolerant strains, it was found the former contained 153 additional genes compared to the latter (Oshone et al., 2017). Seven of these genes were upregulated under saline conditions and were associated with alterations to the cell envelope, changes in membrane fluidity and compatible solute synthesis (Oshone et al., 2017).

Aside from the genes exhibited only by the highly salt tolerant strains, salt tolerance in *Frankiaceae* is also thought to occur through several other mechanisms including exclusion and removal of salt from the cell through the action of P‐type ATPases, Na+/H+ antiporters and Na+‐ATPases. This is exemplified by the cells of *Frankiaceae* strains Ceq1 and Ema1, which were observed to have intracellular NaCl concentrations less than 30 and 20 mM, respectively, despite the surrounding media containing 500 and 200 mM NaCl, respectively (Oshone et al., 2017; Tani & Sasakawa, 2000; Tani & Sasakawa, 2003). It was noted that increased NaCl concentrations raised the rate of cellular sodium efflux, indicating that active efflux is engaged to remove salts from the cell (Srivastava et al., 2012).

Furthermore, Ghedira et al. (2017) demonstrated in *F. alni* that ABC transporters, are upregulated in response to desiccation, which maintains osmotic balance by allowing movement of ions. Interestingly, the mechanosensitive ion channel MscL was upregulated in response to desiccation, which opens in response to mechanical changes in the cell membrane, preventing osmotic damage (Ghedira et al., 2017). Usually, such channels open in response to a hypotonic environment, but this channel may be upregulated to prevent overaccumulation of compatible solutes, which could result in cell lysis (Booth & Louis, 1999).

To further facilitate salinity tolerance in *Frankiaceae* several genes, such as glycosyl transferases, nucleoside polysaccharide deacetylases and sugar epimerases, are upregulated to reduce salt influx through cell envelope modification (Oshone et al., 2017). This was corroborated by Ghedira et al. (2017), as the most upregulated *F. alni* gene in response to desiccation was a putative autotransporter adhesin, which may play a role in cell envelope remodelling. Under salt stress the *Frankiaceae* cell membrane may also be modified to become more fluid, to better resist salinity imposed osmotic stress. This modification is driven by upregulation of enzymes such as acyl‐acyl carrier protein desaturases, which converts saturated fatty acids to unsaturated fatty acids, and upregulation of ubiquinone biosynthesis to increase mechanical stability and fluidity of the membrane (Oshone et al., 2017).

Another method employed by *Frankiaceae* to improve salinity tolerance is upregulated synthesis of compatible solutes to reduce osmotic water loss from the cell (Oshone et al., 2017) (Figure 1). This occurs through several mechanisms, with genes facilitating synthesis of *N*‐acetylglutaminylglutamine amide, upregulated synthesis of trehalose and upregulated production of glutamate (Ghedira et al., 2017; Oshone et al., 2017). In addition, the action of unregulated peptidases and proteases indirectly result in the release of amino acids, which act as compatible solutes (Ghedira et al., 2017).

To further protect the cell from osmotic damage, several reactive oxygen species scavenging genes and DNA repair genes are also upregulated (Ghedira et al., 2017). In tandem, three major groups of proteins are downregulated in response to salinity, with these genes involved in nitrogen fixation, respiration and homologous recombination. These are likely downregulated as they facilitate highly energy demanding processes that are not essential to immediate cellular survival (Ghedira et al., 2017).

Drawing upon the few examples within the literature, inoculation of actinorhizal plants with saline tolerant *Frankiaceae* leads to increases in actinorhizal salinity tolerance and seems a feasible method to improve performance in this regard. Ngom et al. (2016) showed that inoculation of *C. glauca* with *Frankiaceae* strains CcI3 and CeD increased the growth, biomass and chlorophyll content of shoots across all tested salinity ranges (0–500 mM) compared to uninoculated plants. Additionally, strain CcI3 increased root biomass compared to uninoculated *C. glauca* plants in all saline concentrations and inoculation with strain CeD increased root biomass in NaCl concentrations greater than 100 mM. This increased salinity tolerance may be due to accumulation of the osmoprotectant proline, with proline concentration increasing at all salinity concentration in *C. glauca* plants inoculated with strains CcI3 and CeD compared to uninoculated plants (Ngom et al., 2016) (figure 2.1).

However, there are also examples illustrating more modest improvements in actinorhizal salinity tolerance when inoculated with *Frankiaceae*. For example, *C. equisetifolia* inoculated with *Frankiaceae* strain CeD increased shoot growth, total biomass and chlorophyll content only in NaCl concentrations lower than 200 mM (Ngom et al., 2016). Likewise, *C. glauca* Sieb. ex Spreng inoculated with *Frankiaceae* sp. Thr did not show increased survival in saline conditions compared to uninoculated plants supplied with KNO_3_ (Batista‐Santos et al., 2015; Duro et al., 2016). Such differences may arise due to intraspecific variation between *Frankiaceae* associated with differing actinorhizal species, demonstrating why it is useful to investigate a range of *Frankiaceae* for differing plants.

### Frankiaceae*‐actinorhizal tolerance to heavy metals and reduction of soil heavy metals*

Actinorhizal plants possess several characteristics that make them suited to bioremediation of heavy metal polluted soils (Table 2), with the ability of *A. glutinosa* to tolerate high concentration of high metals being well documented (Figure 1). For example, 92% of these plants survived after two growing seasons when planted upon heavy metal contaminated saline river dredgings, with this being 21% higher than other pioneer species such as White Poplar (*Populus alba* L.) (Mertens et al., 2004).

A further example of such tolerance was observed when growing *A. glutinosa* and *Alnus incana* upon copper and lead polluted soil derived from a copper smelter. While under metal stress, leaf dry mass was significantly reduced in both species, however, there was no significant reduction in stem, root and nodule dry biomass or total seedling biomass (except in the case of Krzyż derived *A. incana*) (Lorenc‐Plucińska et al., 2013). In a similar manner to actinorhizal plant performance, *Frankiaceae* symbiosis and nitrogen fixation is reduced under heavy metal stress, but not completely inhibited (Bélanger et al., 2015; Lorenc‐Plucińska et al., 2013). This demonstrates that while growth and *Frankiaceae* symbiosis are somewhat decreased under metal contaminated soils, *A. glutinosa* and *A. incana* are still capable of growth under these conditions in association with *Frankiaceae*. It is probably that such tolerance is not exclusive to *Alnus*, as many actinorhizal species are found upon metal polluted sites.

Studies such as Wheeler et al. (2001) challenge the notion of actinorhizal plants being metal tolerant, whereas *A. glutinosa* was negatively impacted by nickel concentrations as low as 0.225 mM. However, it should be noted that this investigation was conducted under hydroponic conditions, with a highly soluble form of nickel (NiSO_4_.6H_2_O). Thus, this may result in greater nickel bioavailability than is typical within soil, increasing the toxicity of nickel to plants by allowing more nickel to be taken up than usual. This may explain why the findings of this study differ from the majority of the other literature regarding actinorhizal plants metal tolerance.

In addition to their tolerance to heavy metals, actinorhizal plants are well documented as metal excluders (Ghazouani et al., 2020; Jing et al., 2014; Rosselli et al., 2003). Metals taken up by the plants are stored in root tissue of these species, with some metals such as zinc and nickel being sequestered in the nodules specifically (Callender et al., 2016; Lorenc‐Plucińska et al., 2013; Wheeler et al., 2001). This nodule metal sequestration may be due to the production of metallohistins by *A. glutinosa* which are proteins that sequester a range of metals (zinc, nickel, cobalt, copper, cadmium, and mercury) in the nodule, possibly for use in *Frankiaceae* metabolism (Gupta et al., 2002; Pawlowski et al., 1997).

Examples of such metal exclusion is evident in multiple studies investigating is *A. glutinosa* grown upon heavy metal polluted soils. With these plants presenting foliar heavy metal concentrations within the normal range for cadmium, copper, lead, zinc, magnesium, and iron despite the metal polluted conditions (Callender et al., 2016; Desai et al., 2019; Mertens et al., 2004; Vandecasteele et al., 2008). Nevertheless, there is some discrepancy between these studies, as some certain metals show elevated foliar concentrations compared to control plants. However, such differences likely arise from variability in the amounts of metals present and the particular chemistry of the soil at the differing study sties and thus do not detract from the notion of these plants being metal excluders. Also in some instances, the level of foliar translocation of these metals decreased over time, as seen in the case of lead in Desai et al. (2019), likely due to growth in root biomass allowing greater metal storage.

This ability of certain plants to exclude metals from their aerial tissue is desirable for bioremediation, ensuring metals are not taken up into the leaves and recycled back into the soil in high amounts during leaf senescence (Figure 1). Aside from preventing metal accumulation in the soil, this also prevents metals becoming more bioavailable and mobile as they will not be released alongside the metal complexing dissolved organic matter released during leaf litter decomposition (Antoniadis & Alloway, 2002; Kügler et al., 2019; Küsel & Drake, 1998; Mertens et al., 2004; Mohr et al., 2022; Robinson et al., 2000; Shahid et al., 2013; Strobel et al., 2001).

Regarding application to bioremediation of heavy metal polluted sites, *A. glutinosa* was planted upon the former coal mine in Varteg, Wales. Over the course of 14 years, soil metal levels were significantly reduced compared to unplanted sites, with these reductions ranging from 35 to 52% across a range of metals (Desai et al., 2019). An additional example was the plantation of *A. glutinosa* and *A. incana* grown upon copper and lead polluted soil from a copper smelter (Lorenc‐Plucińska et al., 2013). Likewise, *Alnus hirsute* and *Alnus firma* have been shown to decrease the concentration of a range of metals when planted at high or low density (Lee et al., 2009). However, it was noted in this study that leaching due to rain contributed to more metals being removed from the soil than the plants, but it is likely that this would decrease over time as larger root systems develop to facilitate metal uptake (Lee et al., 2009).

*Frankiaceae* is a well‐documented family of heavy metal tolerant bacteria (Table 2). The majority of *Frankiaceae* species have been described as possessing some degree of resistance to metals including lead, chromium and selenium, with slightly lower resistance reported to silver, cadmium, antimony and nickel, with copper tolerance varying between 2 and 20 mM dependent upon the species (Richards et al., 2002). Tolerance to other metals may depend upon their chemical form, as seen in the case of arsenic, where tolerance to AsO_4_^3−^ was greater than AsO_2_^1−^ (Richards et al., 2002).

Heavy metal tolerance in *Frankiaceae* may occur through several mechanisms, with chemical modification of the metal to a less toxic form and removal of metals from the cells being the primary methods. This is illustrated by the reduction of selenite to selenium via an NADH dehydrogenase after selenite is exported from the cell (Rehan et al., 2019), the reduction of arsenate to arsenite by a thioredoxin‐dependent phosphotyrosine‐phosphotase (ArsC2) and the presence of a cadmium‐inducible glyoxylase (Furnholm & Tisa, 2014). In addition to reduction, precipitation is used as a means of chemical modification of metals, for example, undecaprenyl phosphatase and DUF347 (unknown function) causes the precipitation of lead on the *Frankiaceae* cell surface after exportation from the cell by CopA (Furnholm & Tisa, 2014). Additional examples of metal removal from the *Frankiaceae* cell include the CzcD transporter, which is involved in export of zinc, cadmium, copper, and arsenic (Furnholm & Tisa, 2014) and the P‐type ATPases, CopA, which serves to remove copper (Rehan, Furnholm, et al., 2014) (Figure 1).

Other detoxification mechanisms present within *Frankiaceae* involve the use of chaperones to ensure metals are correctly transported to the sites of metabolism, export and chemical modification. The cobalt chaperones CobN and CbiX, are examples which serve to protect against the toxic effects of cobalt (Furnholm & Tisa, 2014) (Figure 1).

The above detoxification methods will render the metals having reduced mobility and toxicity, increasing the survivability of the host plant and the *Frankiaceae*, with metal uptake reducing soil metals over time. Aside from the aforementioned detoxification mechanisms, some *Frankiaceae* (such as sp. CH37) are capable of producing metallophores which allow selective exclusion of metals (Deicke et al., 2019; Kraemer et al., 2015; Mohr et al., 2021). This is demonstrated by Frankobactin A1, which binds copper with high affinity preventing its entry into the cell and therefore increasing *Frankiaceae* resilience to copper (Mohr et al., 2021). This has also been observed in other species such as *Azotobacter vinelandii*, which use a similar metallophore system to selectively take up molybdenum while blocking tungsten uptake (Wichard et al., 2008).

In contrast to the beneficial attributes of *Frankiaceae* regarding metal pollution, it has been proposed that *Frankiaceae* nitrogen fixation may lead to a decrease in soil pH, increasing metal mobility (Bolan et al., 1991; Mertens et al., 2004; Pavlů et al., 2021; Tang et al., 1999; Wang et al., 2015). However, over the course of 7 years the pH of soil under an *A. glutinosa* stand did not substantially decrease in pH compared to the soil beneath the non‐nitrogen fixing Silver Birch (*Betula pendula* Roth.) and Scots Pine (*Pinus sylvestris* L) (Kuznetsova et al., 2011). Additionally, this rate of acidification (if occurring at all) would likely decrease over time, as nitrogen fixation would slow as soil nitrogen increases (Mertens et al., 2004; Tang et al., 1999).

As *Frankiaceae* display the ability to detoxify metals, inoculation of actinorhizal plants with *Frankiaceae* has been linked to improvements in plant performance upon contaminated sites (Table 2). An example of this is the inoculation of *C. equisetifolia* with *Frankiaceae*, which, in comparison to uninoculated plants, led to significantly increased plant height, collar diameter, cladophyll biomass and number of branches, both 3 months and 2 years after planting upon bauxite mines spoil (Karthikeyan et al., 2009). In addition, uninoculated plants showed only 35% survival after 2 years, whereas 90% of the *Frankiaceae* inoculated plants survived. Likewise, inoculation of *A. glutinosa* with *F. alni* resulted in increases in total dry weight, root dry weight and shoot dry weight, compared to uninoculated plant, in soils contaminated with Cu, Ni, Zn, Pb, or Cd (Bélanger et al., 2015).

Despite the benefits conferred by *Frankiaceae* inoculation, only a small number of studies specifically utilizing *Frankiaceae* inoculated plants for bioremediation of heavy metal polluted sites have been reported. These are exemplified by the bioremediation of mine tailings from Val‐d'Or, Quebec, Canada using Frankia inoculated *A. glutinosa* and *Alnus crispa* (Callender et al., 2016). Here, these *Frankiaceae* inoculated plants were able to significantly lower nickel, chromium, barium and cobalt in both bulk and rhizospheric soil compared to unplanted soil, with the largest decrease being an 84.8% reduction in chromium in *A. glutinosa* bulk soil (Callender et al., 2016). It was also noted that the levels of copper and sodium were higher in the rhizospheric and bulk soil of planted sites compared to unplanted sites (Callender et al., 2016).

### Frankiaceae*‐actinorhizal pollution degradation*

A number of differing pollutants are degraded by *Frankiaceae* (Figure 1) (Table 2), In the case of atrazine, *Frankia alni* has demonstrated capability to grow upon media containing atrazine as the sole carbon and nitrogen source, indicating an ability to degrade atrazine (Rehan, Kluge, et al., 2014). Atrazine metabolism is proposed to occur due to *trzN*, *atzB*, and *atzR* genes which encode an amidohydrolase, an adenosine aminohydrolase and a LysR‐type transcriptional regulator, respectively (Rehan et al., 2016; Rehan, Kluge, et al., 2014). The TrzN enzyme dechlorinates atrazine by hydrolysing the C‐Cl bond, producing hydroxylatrazine, which is dealkylated by AtzB to produce N‐isopropylammelide (Martinez et al., 2001). This N‐isopropylammelide is then metabolized through four hydrolysis reactions, conducted by AtzCDEF, producing 2CO_2_ and 2NH_3_ (Martinez et al., 2001). Some *Frankiaceae* such as *Pseudofrankia inefficax* EuI1c, contain the *trzN*‐*atzBR* and *atzCDEF* clusters, indicating potential to carry out complete degradation of atrazine. Other species such as *F. alni*, while capable of atrazine degradation in vitro, lack the *atzC* and *atzD* genes (Martinez et al., 2001; Rehan, Kluge, et al., 2014). This indicates that varying degrees of biodegradation of atrazine could be present within *Frankiaceae*, with some species completely mineralising atrazine and others producing intermediates. Alternatively, this could also suggest that there are potentially unknown genes that could conduct an analogous function to *atzCD*.

According to genomic studies, in *Frankiaceae* has the potential to degrade biphenyl compounds such as PCB and dioxins. It is proposed that biphenyls are degraded to 2‐hydroxypenta‐2,4‐dienoate and benzoate using the *bphABCD* operon through the biphenyl upper meta‐cleavage pathway. The resulting 2‐hydroxypenta‐2,4‐dienoate is then further degraded through the lower pathway of aromatic ring degradation using the genes *bphEFG* (also known as *bphHIJ*) to produce acetyl‐CoA (Pieper & Seeger, 2008; Rehan et al., 2016). In *Frankiaceae* genomes, annotation of the *bphA1A2A3BCDEFG* and *bphBCDEFG* gene clusters is relatively frequent, having been previously reported in strains EuI1c, EUN1f, CcI49, G2, and R43 (EuI1c lacks *bphA3* and *bphH*) (Mansour et al., 2017; Pieper & Seeger, 2008; Rehan et al., 2016). This would indicate ability to metabolize these pollutants and reduce the exposure risks associated with them; however, the functionality of these has not yet been confirmed in vitro.

Additionally actinorhizal plants have been shown to effectively grow upon hydrocarbon polluted sites, with species such as *A. glutinosa* demonstrating a 93% survival rate when planted on open cast oil shale mining areas (Kuznetsova et al., 2010). Furthermore, when planted upon hydrocarbon polluted sites, actinorhizal plants including *Alnus viridis*, *A. incana*, and *C. equisetifolia* have reduced concentrations of soil hydrocarbons (Bissonnette et al., 2014; Kuznetsova et al., 2010; Lefrançois et al., 2010; Sun et al., 2004). These decreases are largely attributed to microbial activity, as *Frankiaceae* demonstrates the ability to degrade hydrocarbons.

It is proposed that *Frankiaceae* uses the protocatechuate pathway to degrade the polyaromatic hydrocarbon naphthalene (and derivatives) into acetyl‐CoA and succinyl‐CoA, via ortho‐cleavage (Baker et al., 2015; Rehan et al., 2016). The protocatechuate pathway used in such degradation in *Frankiaceae* QA3 is composed of eight genes encoding: a protocatechuate 3,4 dioxygenase alpha and beta subunits, fumarate lyase (3‐carboxy‐cis,cis muconate cycloisomerase), 4‐carboxymuconolactone decarboxylase/3‐oxoadipate enol lactonase, 4‐hydroxybenzoate 3‐monooxygenase, oxoacid‐CoA transferase alpha and beta subunits and phthalate 4,5 dioxygenase (Baker et al., 2015; Rehan et al., 2016). This is similar to the gene cluster found in *Rhodococcus opacus* 1CP and *Rhodococcus ruber* OA1, which also degrade naphthalene (Eulberg et al., 1998; Li et al., 2016; Rehan et al., 2016). The first five genes of this cluster are also found in *Frankiaceae* EuI1c and EUN1f, indicating these strains can also potentially degrade naphthalene. Furthermore, *Frankiaceae* may also be able to degrade alkanes found in hydrocarbon fuels, as both *Frankiaceae* ACN14a and EAN1pec are known to contain the alkane‐1 monooxygenase (*alkB*) gene (Rehan et al., 2016). With this gene also present in *Rhodococcus* Q15 and *Rhodococcus* NRRL B‐16531, both of which have been shown to utilize *alkB* to degrade C12‐C16 alkanes (Whyte et al., 2002).

Phenol compounds are another class of pollutants which *Frankiaceae* may be able to degrade. It is thought phenols are degraded by catechol‐2,3‐dioxygenase, which is used in meta‐degradation of phenols in other bacterial species, including the closely related *Rhodococcus* (Ali et al., 1998; Arif et al., 2011; Hughes et al., 1984; Rehan et al., 2016). Furthermore, *Frankiaceae* alters production of host plant root exudates to increase production of phenol compounds, as these may be metabolized by *Frankiaceae* (Popovici et al., 2011). Conversely phenolic compounds have been shown to inhibit growth of some *Frankiaceae*, although strains such as AvcI1 were less inhibited by certain phenols such as caffeic acid, with strains EuI1b and Pt410 seeming to display increased growth as the concentrations of o‐hydroxyphenylacetic acids increased (Vogel & Dawson, 1986). This suggests that some *Frankiaceae* can degrade certain phenols, with this possibly being due to the host actinorhizal plant they associate with, as suggested in Vogel and Dawson (1986). Further investigation is warranted to determine the ability of the *Frankiaceae*‐actinorhizal symbiosis to degrade phenols, as no major studies have been conducted in this area.

### Frankiaceae*‐actinorhizal mediated soil fertility improvements and* Frankiaceae *plant growth promotion abilities*

*Frankiaceae* is a well‐documented family regarding its plant growth promotion ability and capabilities (Table 2). *Frankiaceae* are noted nitrogen fixing bacteria, with the nitrogenase encoding *nif* operon found in *Frankia*, *Protofrankia*, and *Parafrankia*, but lacking in *Pseudofrankia* (Normand et al., 2018; Nouioui, Cortés‐albayay, et al., 2019; Nouioui, Ghodhbane‐Gtari, et al., 2019) (Figure 1). Based upon the result of Koirala and Brözel (2021), *Frankiaceae* contain a molybdenum‐iron nitrogenase as opposed to a vanadium or iron‐only nitrogenase. Molybdenum within soil is noted as often being a rare element due to its highly soluble nature, however it is noted that tannins, which are produced by actinorhizal plants such as *Alnus*, can bind molybdenum preventing molybdenum being leached from the soil (Wichard et al., 2009). As noted by Deicke et al. (2019) the *Frankiaceae* analysed in their work did not seem to contain a molybdenum chelating system to extract tannin bound molybdenum. Thus, *Frankiaceae* may use an as of yet undiscovered molybdenum chelating agent, rely on host plant molybdenum uptake, or use catechol type siderophores to facilitate molybdenum uptake into the nodules (Arahou et al., 1998; Bellenger et al., 2008; Pourhassan et al., 2015).

Nevertheless, *Frankiaceae* symbiotic association with actinorhizal plants allows these plants to take up fixed nitrogen, in the form of ammonia, with this added to soil through decomposition of plant material (Izquierdo et al., 2005; Khamzina et al., 2009; Mailly & Margolis, 1992; Parrotta, 1999; Qi et al., 2018; Tarrant et al., 1969; Wall, 2000; Zavitkovski & Newton, 1968). As demonstrated by *A. incana* leaf litter, this rich material may aid in the decomposition and nutrient release of other, less degradable material from species such as *Populus tremuloides*, further improving soil fertility (Taylor et al., 1989). This general improvement in decomposition is likely driven by the nitrogen rich actinorhizal leaf litter facilitating faster growth of saprotrophic organisms which break down organic material (Taylor et al., 1989). Alongside nitrogen fixation, *Frankiaceae* have also been shown to produce ammonia, which can further aid in plant growth as a nitrogen source (Marappa et al., 2020).

In addition to nitrogen fixation, most *Frankiaceae* genomes are noted as containing enzymes involved in phosphate solubilization, such as alkaline phosphatase and phosphodiesterase/alkaline phosphatase D, allowing for improved phosphorus uptake in host plants (Nouioui, Cortés‐albayay, et al., 2019) (Figure 1). In addition, several other genes possibly potentially involved in phosphate solubilization, such as *ptsABCS*, *phoBHRU*, and various phosphate transporters (*lat*, *pho*), appear to be present in several *Frankiaceae* genomes (Nouioui, Cortés‐albayay, et al., 2019). This was corroborated by in vitro investigations that have demonstrated the ability of *Frankiaceae* to solubilize inorganic phosphates sources in media (Marappa et al., 2020). Unsurprisingly, actinorhizal growth in field conditions has been shown to improve soil phosphate, likely due to association with *Frankiaceae* (Qi et al., 2018; Uri et al., 2001).

Siderophore production is another useful trait to aid plant growth and improve soil nutrients, as siderophores chelate iron increasing its bioavailability. As such, plants inoculated with siderophore producing bacteria show greater growth than uninoculated plants on soils that are rich or poor in iron (Rungin et al., 2012). Based upon genomic evidence, the majority of *Frankiaceae* contain siderophore producing clusters, with production of siderophores in some strains confirmed during in vitro screening (Marappa et al., 2020; Nouioui, Cortés‐albayay, et al., 2019) (Figure 1). Nevertheless during in vitro studies, some *Frankiaceae* do not produce siderophores, despite containing the genes to produce siderophores. This could possibly be due to these strains lacking the necessary substrates or environmental cues to initiate siderophores production, as commonly seen in production of other secondary metabolites such as antibiotics (Sánchez et al., 2010). Thus, siderophore production in these strains should not be discounted entirely and should be investigated further under differing conditions.

As an alternative to siderophores, some *Frankiaceae* strains such as strains AvsI3 and HFPCpI1, appear to instead secrete oxalic acid to solubilize iron under in vitro conditions (Arahou et al., 1998; Aronson & Boyer, 1994). In comparison to a range of other organic acids tested, oxalic acid has been shown to be highly effective in solubilizing metals, including iron (Ambikadevi & Lalithambika, 2000; Nworie et al., 2017).

Antibiotic production is another useful characteristic of *Frankiaceae*, potentially aiding in disease suppression to improve plant growth. *Frankiaceae* strains such as AiPs1 produce the antibiotic Demethyl (C‐11) cezomycin, originally known as Frankiamide (Haansuu et al., 2001; Klika et al., 2001; Klika et al., 2003). Demethyl (C‐11) cezomycin has demonstrated activity against a range of microorganisms, including 14 Gram positive bacteria and six fungal species, with such activity thought to be due to inhibition of cellular calcium flux (Haansuu et al., 2001). Further genome analysis suggests that *Frankiaceae* ACN14, CcI3 and EAN1pec could exhibit high potential to produce a range of other antibiotic compounds, with 65 biosynthetic clusters identified between these three strains alone, with many of these appearing to encode unique products (Udwary et al., 2011).

*Frankiaceae* genomes are also rich in genes that potentially encoding lytic enzymes including chitinases, cellulases, endoglucanases, and extracellular endoglucanases (Nouioui, Cortés‐albayay, et al., 2019). The production of such lytic enzymes by *Frankiaceae* has been demonstrated to confer antimicrobial activity in vitro against fungi and bacteria such as *Pseudomonas* and *Colletotrichum* (Marappa et al., 2020; Nouioui, Cortés‐albayay, et al., 2019; Safo‐Sampah & Torrey, 1988). Furthermore, it was reported that *C. equisetifolia* plants were more resistant to *Rhizoctonia solani* infection when inoculated with *Frankiaceae* UMCe12 conferring up to 81.1% overall disease resistance (Gopinathan, 1995).

*Frankiaceae* may also directly influence host plant growth by the production of phytohormones. The genomes of several *Frankiaceae* contain the genes encoding anthranilate synthase and aminase component, indole‐3‐glycerol phosphate synthase and anthranilate phosphoribosyltransferase, which allows production of anthranilate, an indole precursor, which is in turn a precursor of indole‐3‐acetic acid (Di et al., 2016; Nouioui, Cortés‐albayay, et al., 2019). However, it is unknown if the *Frankiaceae* contain any of the other genes in the five bacterial pathways that allow synthesis of indole‐3‐acetic acid (Di et al., 2016). Despite this lack of genome analysis, species such as *Frankiaceae* sp. DDNSF‐01, *Frankia casuarinae*, *Frankiaceae* sp. AvcI1 and *Parafrankia discariae* BCU110501 have demonstrated ability to synthesize indole‐3‐acetic acid under in vitro conditions (Marappa et al., 2020; Solans et al., 2011; Wheeler et al., 1984).

When the composition of indole compounds produced by *Frankiaceae* HFPArI3 were analysed, the most abundant were indole‐3‐ethanol (25.4 ng ml^−1^) and indole‐3‐acetic acid (8.4 ng ml^−1^) (Berry et al., 1989). However, these levels were only achieved when exogenous tryptophan was supplied (50 μM). The need for exogenous tryptophan may be indicative of these processes only being upregulated under specific conditions which may be provided by a plant host. In terms of function, increasing concentration of indole‐3‐ethanol applied to the root zone have been found to increase the number of lateral roots produced (Berry et al., 1989). Indicating this compound, despite not being the “classical auxin” can still improve plant growth. Interestingly Berry et al. (1989) observed that the ethyl acetate partitioning step used in analysis reduced indole‐3‐ethanol levels, suggesting this may be a more common constituent of indole compounds produced by PGP bacteria than initially noted.

Aside from indole compounds, *Frankiaceae* genomes are also characterized by the presence of a conserved biosynthetic cluster containing 11 genes related to the synthesis of cytokinins (Nouioui, Cortés‐albayay, et al., 2019). Production of cytokinins was observed by Stevens and Berry (1988), with *Frankiaceae* sp. HFPArI3 producing N6‐(Δ2‐isopentenyl) adenosine in vitro, with this likely being converted to zeatin cytokinins. Due to contaminating media components, this could not be definitively inferred from the analysis undertaken, it is possible that other cytokinins such as trans‐zeatin riboside and cis‐zeatin riboside were instead produced. More recent study of *P. discariae* BCU110501 has been reported to produce zeatin in vitro, supporting the notion of zeatin cytokinins being produced by *Frankiaceae* (Solans et al., 2011).

*Frankiaceae* genomes also present 1‐aminocyclopropane‐1‐carboxylic acid (ACC) deaminase encoding genes in all species except *F. casuarinae* strains (Nouioui, Cortés‐albayay, et al., 2019). ACC deaminase is a highly desirable enzyme, as its presence allows the bacteria to reduce ethylene levels in the plant, promoting growth and resistance to a range of abiotic stressors including drought, salinity and metal pollution (Glick, 2014). However, the functionality of this enzyme within *Frankiaceae*, has not been investigated *in planta* and in vitro, so it cannot be confirmed definitively if this enzyme promotes the actinorhizal growth. Finally, *Frankiaceae* may also be able to produce gibberellic acid, with this being evident in *P. discariae* BCU110501 under in vitro conditions (Solans et al., 2011). Likewise, this also deserves further investigation to determine both the nature of the gibberellin and the ability of this to improve actinorhizal growth.

## FUTURE DIRECTIONS

### *Deeper insights into* Frankiaceae *and optimization of an endophyte inoculation system*

*Frankiaceae*‐actinorhizal symbiosis offers a fertile area for future study, particularly regarding the bioremediation potential of this association. Work to select optimal *Frankiaceae* isolates for each bioremediation task would be useful, as different *Frankiaceae* vary in terms of their ability to degrade pollutants and their tolerance to salinity and metals (Oshone et al., 2013; Oshone et al., 2017; Richards et al., 2002; Tani & Sasakawa, 2003). In addition, other endophytes able to cooperatively aid in bioremediation should be studied alongside *Frankiaceae*, as these could be used to further bolster their performance. Examples of this include inoculation of *A. glutinosa* with *Glomus intraradices* and *Frankiaceae*, which lead to improved plant performance in a number of metrics than either isolate alone (Oliveira et al., 2005). This has also been demonstrated as co‐inoculation of *Alnus cordata* with *Frankiaceae* and either *Glomus mosseae* or *Glomus fasciculatum* produced significantly greater plant growth (after 1 year on mine spoils), than either inocula in isolation (Lumini et al., 1994).

After a desirable *Frankiaceae* isolate (possibly alongside other endophytes) has been found, it may be possible to increase the ability of that strain to infect the host through use of a carrier system. It has been demonstrated that nodulation of the host plant can be relatively low, with Markham (2005) demonstrating that only 45.9% of *A. incana* developed root nodules after 6 weeks when inoculated with *Frankiaceae*. Carrier systems have been used to enable easier handling of the bioinoculant, promoting their long‐term storage and effectiveness. An example is the encapsulation of *Frankiaceae* in alginate, which has been shown to be effective even after storage at room temperature for 2 years (Frioni et al., 1994). Plants inoculated with alginate‐entrapped *Frankiaceae* showed the expected improvements in nodulation and growth compared to uninoculated plants, demonstrating that the carrier system did not impede the plant growth promoting abilities of *Frankiaceae* (Sougoufara et al., 1989). Further exploration of more sophisticated formulations and application methods, adapted to the actinorhizal‐*Frankiaceae* symbiosis is necessary to maximally utilize this symbiosis at large scale.

### Frankiaceae *genetic modification*

Aside from studying naturally occurring *Frankiaceae*, future research efforts could explore genetic modification of a desirable *Frankiaceae* species or strain to further improve its bioremediative capabilities. As of yet, stable transformation of *Frankiaceae* has proved difficult, one reason being that *Frankiaceae* have no identified phages which could be utilized their genetic modification. It is likely that research in this area could prove fruitful as the genomes of *Frankiaceae* are known to contain integrated phages (Normand et al., 2007). However, as *Frankiaceae* do not form lawns very well on solid media, this could prove a barrier to locate phage plaques (Normand & Lalonde, 1986).

Actinobacteria also have low rates of homologous recombination, most likely due to use of non‐homologous end joining instead of homologous recombination in DNA repair. However, as demonstrated by Zhang, Chen, et al. (2012) in *Streptomyces avermitilis*, when the bacterial homologues of *ku70* and *ku80* are successfully knocked out, the use of non‐homologous end joining is inhibited. This knowledge could potentially be applied to *Frankiaceae* to increase the success of genetic transformation.

An additional factor that may pose a barrier to *Frankiaceae* transformation is the presence of restriction enzymes which may degrade introduced plasmids. This degradation is due to plasmids often being methylated by the enzyme *Dam* after replication (Barras & Marinus, 1989). However, some actinobacteria possess a system which cleaves methylated DNA using type IV restriction enzymes (Janulaitis et al., 1992; Kieser & Hopwood, 1991; MacNeil, 1988; Sutherland et al., 1992). Furthermore, some actinobacteria methylate DNA in a different pattern to bacteria using the *Dam* enzyme, also possessing a DNA mismatch repair pathway that lacks the *Dam* associated DNA repair enzymes, MutS and MutL, with this mismatch repair pathway being more akin to those found in Archaea (Castañeda‐García et al., 2017; Ishino et al., 2016).

Based upon the aforementioned, it is possible that use of unmethylated plasmids could increase transformation efficiency in *Frankiaceae*. This has been previously demonstrated in both *Corynebacteria* and *Streptomyces*, where unmethylated plasmids are not cleaved by the type IV restriction enzymes (Ankri et al., 1996; Kieser & Hopwood, 1991). Gifford et al. (2019) transformed *F. alni* using the unmethylated plasmid pIGSAF, with this stably maintained in *F. alni* for up to 3 weeks in the absence of any selective pressure. Similarly, Pesce et al. (2019) stably transformed multiple *Frankiaceae* strains using a conjugation system, inserting a putative salt tolerance gene from the salt tolerant *Frankiaceae* sp. CcI6 into the salt sensitive *F. casuarinae* CcI3, increasing its performance under saline conditions. The method utilized by Pesce et al. (2019) involved conjugation with a methylation positive *Escherichia coli*, where it is likely that use of a methylation negative *E. coli* would have further increased transformation efficiency (Flett et al., 1997; Gifford et al., 2019; Stein et al., 1988).

Another method of *Frankiaceae* transformation that warrants further exploration is the use of *Frankiaceae* derived plasmids. Such plasmids would most likely allow for increased rates of stable transformation, due to these plasmids being native to *Frankiaceae*. An analogous example demonstrating this is the transformation of *Frankiaceae* strain EuI1c using genomic DNA from *Frankiaceae* strain EAN1pec. This introduced antibiotic resistance in *Frankiaceae* strain EuI1c, that was maintained over several generations, even in the absence of selective pressure (Myers & Tisa, 2003). Thus, demonstrating transformation of *Frankiaceae* with *Frankiaceae* derived DNA allows for effective and stable transformation. Several *Frankiaceae* plasmids have been studied such as pFQ31 and pFQ11, which could serve to allow stable insertion of desirable genes into *Frankiaceae* (Lavire et al., 2001; Normand et al., 1985; Normand & Lalonde, 1986; Xu et al., 2002).

Overall, the above research represents strides in *Frankiaceae* genetic modification which may allow for future modification of these beneficial bacteria. Based upon this review, particularly worthy targets of such modification include increasing *Frankiaceae* growth rate, permit growth in a wider array of media, increase tolerance to abiotic stressors and introduce novel pollutant degradation pathways.

### *Increased application of* Frankiaceae*‐actinorhizal symbiosis in other sectors*

Aside from its use in bioremediation, it may also be possible to utilize the actinorhizal‐*Frankiaceae* symbiosis in silvopasture and agroforestry systems to sustainably improve agricultural productivity while reducing the environmental impact of farming. In terms of silvopasture, application of the actinorhizal‐*Frankiaceae* symbiosis would result in reduction in pollutants and salinity, providing an increase in soil nutrients and potentially high quality, high yielding grazing material. In addition, the improved growth of the grazing material may mean less time is required for each pasture to lay fallow between grazing, improving land use efficiency. When the trees are of a mature height they can be cut down and replanted or coppiced for timber to promote new growth, with good rates of carbon sequestration expected as the trees produce more biomass (Jose & Dollinger, 2019).

Regarding agroforestry, the actinorhizal‐*Frankiaceae* symbiosis could be applied to cropping systems such as shade cropping, taungya cropping, and alley cropping. In shade cropping, crops are grown under the tree canopy, protecting the soil from rain erosion and increasing nutrient input through leaf litter. However, in such a cropping system only shade‐tolerant crops could be utilized due to the tree canopy excluding light (Muschler, 2001). In contrast, taungya cropping involves the planting of crops as normal with the desired trees positioned among the crops. These trees are allowed to develop to maturity for timber harvesting, with increasingly shade‐tolerant crops being planted as the canopy develops (Menzies, 1988). Similarly to shade cropping, crops under taungya cropping benefit from increase nutrient input, with the soil protected from rain erosion. Finally, alley cropping involves planting alternate rows of crops and trees or hedgerows, the latter usually being cut prior to planting the former, to reduce shading, with this cut woody material used as organic fertilizer (Akinnifesi et al., 2006). In this case, the actinorhizal trees would act as windbreaks reducing wind erosion, protecting crops from lodging. Where pollution is a concern, the addition of actinorhizal plants to these cropping systems would also enable the removal of contaminants and salts from the soil, thereby increasing crop performance and reducing the risks health associated with produce grown upon polluted sites.

Actinorhizal plants could also be planted upon marginalized land to meet tree planting goals for carbon sequestration. Therefore, freeing land that has been used for these purposes which could instead be utilized for agricultural purposes. Finally, actinorhizal plants, especially those in the *Casuarina* genus which have previously been used in stabilization of coastal regions, could be utilized to protect agricultural areas from coastal wind and water‐based erosion (De Zoysa, 2008; Jayasingam, 2014). Based upon the tolerance of actinorhizal plants to salinity and waterlogging, it is possible that they could also be generally well suited for plantation in riparian and coastal areas, mitigating degradation and erosion pressures common in such areas, while also serving to reduce flooding risks for farmland and coastal/rural communities.

## CONCLUSION

Increasing soil degradation is a global issue that can severely impact food production and human health. The *Frankiaceae*‐actinorhizal symbiosis may be an attractive method to address such soil degradation, as this symbiosis has the potential to reduce soil pollutants and salinity while increasing soil fertility, with low management requirements and minimal economic costs for establishment and maintenance.

Overall, this system should be further studied and optimized to develop inoculants better suited to the context of each bioremediation case than the endemic bacteria of the actinorhizal plant. Additionally, co‐inoculants and carrier systems could be developed to improve bioremediation efficacy and host plant inoculation. Genetic modification of *Frankiaceae* inoculants is an attractive long‐term goal for this symbiosis, as it could allow for levels of bioremediation far surpassing those found in nature.

Beyond improvements to the symbiosis, it should be leveraged more widely, not only for bioremediation of polluted sites but also for shade cropping, Taungya cropping, alley cropping, silvopasture, coastal stabilization, and flood defence. For these and many other applications, this symbiosis currently remains under‐exploited.

## AUTHOR CONTRIBUTIONS

**Ryan Michael Thompson:** Conceptualization; writing – review and editing; writing – original draft; project administration; funding acquisition. **David George:** Writing – review and editing; supervision; funding acquisition. **Maria del Carmen Montero‐Calasanz:** Writing – review and editing; supervision; funding acquisition.

## CONFLICT OF INTEREST STATEMENT

The authors declare no conflict of interest.

## Data Availability

Data sharing is not applicable to this article as no new data were created or analyzed in this study.
